# Ultrasound image intelligent diagnosis in community-acquired pneumonia of children using convolutional neural network-based transfer learning

**DOI:** 10.3389/fped.2022.1063587

**Published:** 2022-11-24

**Authors:** Xiaohui Fang, Wen Li, Junjie Huang, Weimei Li, Qingzhong Feng, Yanlin Han, Xiaowei Ding, Jinping Zhang

**Affiliations:** ^1^ Department of Pediatrics, Shanghai Sixth People’s Hospital Affiliated to Shanghai Jiao Tong University School of Medicine, Shanghai, China; ^2^ Department of Ultrasound Medicine, Shanghai Sixth People’s Hospital Affiliated to Shanghai Jiao Tong University School of Medicine, Shanghai, China; ^3^ Department of Electronic Information, Shanghai Ocean University, Shanghai, China; ^4^ Department of Electronic Information and Electrical Engineering, Shanghai Jiao Tong University, Shanghai, China

**Keywords:** pneumonia, ultrasound, deep learning, transfer learning, pediatrics, lung

## Abstract

**Background:**

Studies show that lung ultrasound (LUS) can accurately diagnose community-acquired pneumonia (CAP) and keep children away from radiation, however, it takes a long time and requires experienced doctors. Therefore, a robust, automatic and computer-based diagnosis of LUS is essential.

**Objective:**

To construct and analyze convolutional neural networks (CNNs) based on transfer learning (TL) to explore the feasibility of ultrasound image diagnosis and grading in CAP of children.

**Methods:**

89 children expected to receive a diagnosis of CAP were prospectively enrolled. Clinical data were collected, a LUS images database was established comprising 916 LUS images, and the diagnostic values of LUS in CAP were analyzed. We employed pre-trained models (AlexNet, VGG 16, VGG 19, Inception v3, ResNet 18, ResNet 50, DenseNet 121 and DenseNet 201) to perform CAP diagnosis and grading on the LUS database and evaluated the performance of each model.

**Results:**

Among the 89 children, 24 were in the non-CAP group, and 65 were finally diagnosed with CAP, including 44 in the mild group and 21 in the severe group. LUS was highly consistent with clinical diagnosis, CXR and chest CT (kappa values = 0.943, 0.837, 0.835). Experimental results revealed that, after k-fold cross-validation, Inception v3 obtained the best diagnosis accuracy, PPV, sensitivity and AUC of 0.87 ± 0.02, 0.90 ± 0.03, 0.92 ± 0.04 and 0.82 ± 0.04, respectively, for our dataset out of all pre-trained models. As a result, best accuracy, PPV and specificity of 0.75 ± 0.03, 0.89 ± 0.05 and 0.80 ± 0.10 were achieved for severity classification in Inception v3.

**Conclusions:**

LUS is a reliable method for diagnosing CAP in children. Experiments showed that, after transfer learning, the CNN models successfully diagnosed and classified LUS of CAP in children; of these, the Inception v3 achieves the best performance and may serve as a tool for the further research and development of AI automatic diagnosis LUS system in clinical applications.

**Registration:**

www.chictr.org.cn ChiCTR2200057328.

## Introduction

Community-acquired pneumonia (CAP) is a common infectious disease in children and the main cause of hospitalization in children. According to the World Health Organization (WHO), CAP is also the leading cause of death worldwide in children under the age of five years (1, 2). Early and timely diagnosis and disease grading have important clinical significance in improving the cure rate of CAP and reducing the fatality rate. At present, the diagnosis and grading of CAP in children rely primarily on medical history, clinical symptoms, physical signs, and related examinations; among these, chest *x*-ray (CXR) and chest CT are important imaging methods (3). However, interpretations of CXR may differ substantially between different observers, while chest CT has disadvantages including larger radiation damage, high cost, and the inability of young children to cooperate. As a result, parents often refuse early imaging. Currently, domestic and foreign guidelines do not recommend chest CT as a routine examination, and CXR is not recommended for typical cases (4, 5). It is therefore urgent to seek another alternative for children with pneumonia, especially in community hospitals. With the development of ultrasound technology, studies have shown that lung ultrasound can accurately diagnose pneumonia in children and is a safe and feasible examination method (6). Ultrasonography has the advantages of being radiation-free, low-cost, and suitable for bedside operation. However, it is also more dependent on the subjective judgment of the operator, making it easy for inexperienced doctors to misdiagnose. At the same time, the increase in work intensity may increase the misdiagnosis rate even among experienced doctors. Applying artificial intelligence (AI) to LUS can make up for the shortcomings of traditional ultrasound operations and improve the diagnosis of CAP in children.

Recent new and exciting advances in the applications of AI in many healthcare areas have inspired innovations in the development of novel AI-based CAP diagnostic technology. Deep learning (DL) is an advanced stage of AI that can simulate the human neural network using a multi-layer neuron cascade learning modus to abstract the original data layer by layer. Features obtained from the data are then exploited for classification prediction. Since DL can learn complex features in images, it has been widely used in medical image analysis. One such method is the use of convolutional neural networks (CNNs) algorithms, which has had remarkable success in medical imaging. CNNs have high potential for feature extraction and analysis and have achieved high precision in CXR of CAP in children. Table 1 lists some relevant studies. Although a comprehensive dataset of ultrasound for CAP in children does not exist, upon the emergence of COVID-19, numerous CNNs techniques have been adopted to diagnose pneumonia of COVID-19. To the best of our knowledge, this is few study that uses CNNs to identify children's pneumonia in LUS images.

**Table 1:** Studies evaluating CNNs used for CAP in children.

Study (country)	Study objective	Image, Population	Models used	Evaluation results
Kermany et al. (China) (7)	Pneumonia detection in children	x-ray, 5,232 images	Inception	Accuracy = 0.96
Rajaraman et al. (America) (8)			VGG 16	Accuracy = 0.96
Sousa et al. (Canada) (9)			VGG19	Accuracy = 0.95
Liang et al. (China) (10)			VGG16, DenseNet121, InceptionV3, Xception, and Proposed method	Accuracy = 0.74, 0.82, 0.85, 0.88, 0.90

In this study, we established a LUS image database to analyze the diagnostic consistency of LUS with clinical manifestations, CXR and CT. At the same time, 8 CNNs—AlexNet, VGG 16, VGG 19, Inception v3, ResNet 18, ResNet 50, DenseNet 121 and DenseNet 201—were constructed to explore the feasibility of ultrasound image diagnosis and grading of CAP in children. The present work is expected to lay a theoretical foundation for the later application of AI technology in clinical LUS diagnosis of CAP in children.

## Objects and methods

### Study design and dataset establishment

A prospective clinical diagnostic study was conducted in a tertiary-level hospital from January 2021 to February 2022. This study was a diagnostic test to evaluate the accuracy of LUS in diagnosing CAP. According to the literature review (26, 27), the estimated sensitivity was 90%, the specificity was 80%, and the allowable error was 0.1. A two-sided test was required, with an alpha of 0.05. Using the formula $N={\left(\frac{Z_{1-\alpha /2}\times \surd p\times (1-p)}{\delta }\right)}^{2}$ to calculate the sample size according to the sensitivity (*n* = 35) and specificity (*n* = 62), the larger value was selected. At least 62 cases should be included in this trial. Eighty-nine patients were recruited in this study finally.

Inclusion criteria: (1) age more than 1 month and less than 14 years; (2) for children over two years of age, a body mass index (BMI) below the 95^th^ percentile of the same age and sex; (3) patients in the experimental group had two or more of the following symptoms: fever, cough, wheezing, increased respiratory rate, dyspnea, three concave signs, nasal fan, and audible moist rales (5); (4) patients in the control group had fever or upper respiratory tract infection symptoms such as cough, nasal congestion, runny nose, and sore throat; (5) consent to complete CXR or chest CT examination; (6) signing the informed consent form and agreeing to be included in the trial.

Exclusion criteria: (1) interval between CXR and LUS exceeding 72 h; (2) interval between chest CT and LUS exceeding 72 h; (3) other serious diseases such as thoracic deformity, congenital heart disease, chest skin trauma or infection, etc., which may affect the study results; (4) patients who could not complete the LUS; (5) patients who had completed the LUS inspection without images; (6) knowing the results of chest x-ray or chest CT and lung ultrasonography.

All patients included in the study completed medical history collection, laboratory examination, chest CT or (and) CXR examination. In order to avoid more radiation, CXR is the first choice, the clinical condition and evolution of patients drove to performance of chest CT. The clinical diagnosis and grading criteria referred to were the “Code of Diagnosis and Treatment of Children with Community-Acquired Pneumonia (2019 Edition)” (5), and the clinical diagnosis was made by experienced clinicians. According to clinical manifestations and chest CT or (and) CXR examination, the children were divided into a non-CAP control group and an experimental group; the experimental group was then further divided into a mild CAP group and a severe CAP group. Lung ultrasonography was performed on all children.

All lung ultrasound examiners were experienced physicians with relevant professional qualifications who had received special training in LUS examination. The Philips Affiniti70 ultrasonic diagnostic apparatus was used for LUS examination; the line was used in infants and preschool children, while array probes (5–12 MHz), and convex array probes (3–5 MHz) were used in children older than six years. The patient was placed in the supine and prone positions in a quiet state (sitting and lateral positions were used to fully expose the chest wall if necessary), and the lungs were divided into 12 regions by the anterior axillary line, the posterior axillary line, and the bilateral nipple lines on the bilateral chest walls, respectively. Each area was scanned horizontally and vertically. According to the relevant guidelines for the diagnosis of CAP in children by LUS at home and abroad (11, 12), the criteria selected in this study were: (1) lung consolidation with air trachea sign or bronchial fluid fill sign; (2) abnormal pleural line and disappearance of A-line; (3) ≥3 B-lines or fused B-lines or dense B-lines; (4) pleural effusion. Normal LUS manifestations were as follows: (1) under B-mode ultrasound, the pleural line and A-line of the normal lung field are both smooth, clear, and regular linear hyperechoic, and arranged in parallel at equal intervals. From the superficial to the deep, the echo of the A-line gradually weakens; (2) <3 B lines; (3) no consolidation and pleural effusion; (4) lung sliding sign exists under real-time ultrasound.

### Pre-processing and augmentation of image dataset

Firstly, images were removed redundant information by cropping and then resized to 513 × 513 pixels. Given the very particular images we are dealing with, we carefully selected data augmentation techniques to diversify the obtained dataset. Random horizontal flip is always adopted in other medical imaging experiments. On the contrary, a vertical flip would yield to a non-sense image where the pleural line is upside-down, thus this operation is not considered. We randomly rotated the image in the range [−10°, 10°] in order to simulate different incidence angles of the probe. A stronger rotation would give an unnatural image; thus, it is avoided (13). Finally, to simulate multiple acquisitions of the same image with multiple devices, and thus make the training procedure robust against different calibrations and instruments, we randomly modified the brightness and the contrast of the images in a relative range of 25% (14).

As such transformations can naturally occur with diverse ultrasound devices and recording parameters, augmentation adds valuable and realistic diversity that helps to prevent overfitting.

### Transfer learning, cross validation and CNN architectures

**Figure 1:**
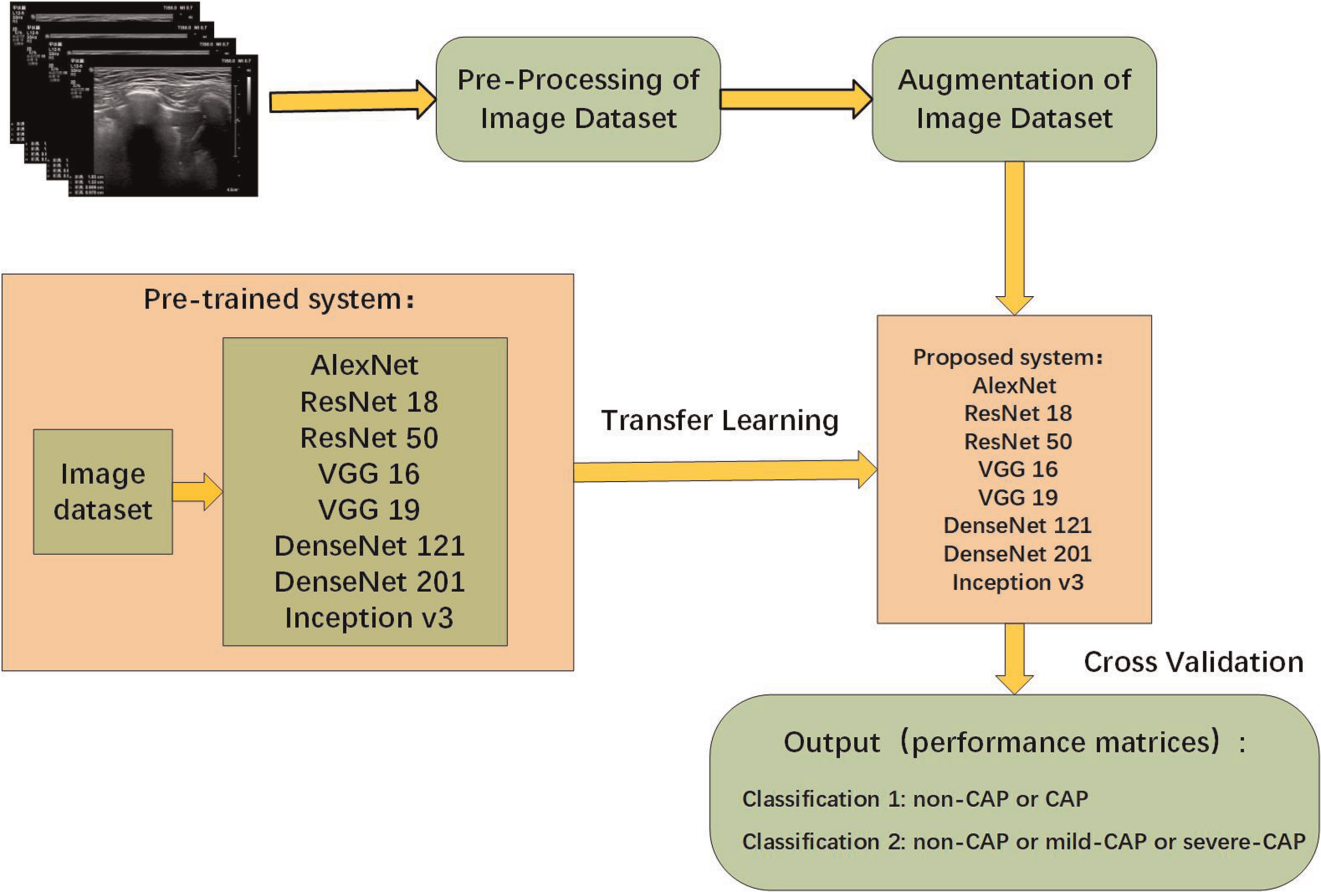
Framework for the detection of CAP and severity classification.

Transfer learning (TL) is the application of knowledge learned from one environment to a new environment. The parameters in the classical CNNs have been trained on ImageNet using millions of images, and the extracted features have been shown to be effective for image classification. TL can solve different tasks simply by fine-tuning the trained model and training it with a small amount of labeled data. TL can also be implemented on ordinary personal computers, without high hardware requirements, and the model training usually only takes a short time to complete. The TL methodology has yielded beneficial and significant achievements in a variety of medical areas (15–17). Owing to these advantages, we utilize 8 pre-trained architectures to deal with the LUS image dataset, rather than using the long training process from scratch. We selected 8 distinct pre-trained CNN architectures: AlexNet, VGG 16, VGG 19, Inception v3, ResNet 18, ResNet 50, DenseNet 121 and DenseNet 201. The proposed methodology was illustrated in Figure 1. It is necessary to set and optimize parameters as the CNN architecture itself is not able to define parameters for the fine-tuning method. The setting of parameters for CNN architectures were shown in Table 2.

**Table 2:** The setting of parameters for CNN architectures.

Parameters	AlexNet	ResNet 18	ResNet 50	VGG 16	VGG 19	Dense-Net 121	Dense-Net 201	Inception v3
Optimizer	ADM	ADM	ADM	ADM	ADM	ADM	ADM	ADM
Base learning rate	0.0001	0.0001	0.0001	0.0001	0.0001	0.0001	0.0001	0.0001
Momentum	0.9	0.9	0.9	0.9	0.9	0.9	0.9	0.9
epochs	30	30	30	30	30	30	30	30
Train batch size	32	32	32	32	32	32	32	32
Test batch size	32	32	32	32	32	32	32	32

Cross-validation is any of various similar model validation techniques for assessing how the results of a statistical analysis will generalize to an independent data set. We selected k-fold cross-validation. The cross-validation process is then repeated k times, with each of the k subsamples used exactly once as the validation data. The k results can then be averaged to produce a single estimation. The advantage of this method over repeated random sub-sampling is that all observations are used for both training and validation, and each observation is used for validation exactly once. Different values of K result in different outcomes, and we select k of 5,8,10 in the study.

### Alexnet architecture

The AlexNet model won the 2012 ImageNet competition by a large margin. The model consists of five convolutional layers and three fully connected layers. The extraction of image feature information is mainly completed by the convolutional layers, while the role of the fully connected layers is to integrate local feature information, flatten the feature information, and then complete the classification task (18).

### VGG 16 and VGG 19 architecture

It was found that the greater the network depth, the stronger the classification function of the network (19). CNN gradually developed from the eight-layer AlexNet model to the 16-layer and 19-layer VGGNet model.

### Inception v3 architecture

The Inception network is an important milestone in the history of CNNs. Before Inception, most CNNs simply stacked more and more convolutional layers, making the network deeper and deeper. Inception performs multiple convolution or pooling operations in parallel and stitches all the output results into a very deep feature map (20). Inception v3 proposes a series of corrections that increase accuracy and reduce computational complexity (21).

### Resnet 18 and ResNet 50 architecture

However, blindly stacking layers cannot improve performance and may even reduce the rate of network convergence. He et al. (22) proposed the ResNet model, which effectively alleviated the problems of gradient disappearance and network degradation. This model can increase the training speed and greatly improve the generalization ability and robustness of deep networks. The ResNet model comprises multiple residual units, each of which consists of a convolutional layer, a batch normalization layer, and a ReLU function. With the increasing number of residual units, models such as ResNet-18 and ResNet-50 have been developed.

### Densenet 121 and DenseNet 201 architecture

The DenseNet model, its basic idea is the same as ResNet, but it establishes a dense connection between all the previous layers and the latter layers (23). Another major feature of DenseNet is feature reuse through the connection of features on channels. These features allow DenseNet to achieve better performance than ResNet with fewer parameters and computational costs.

### Statistical analysis and performance matrices

The SPSS 22.0 software package was used for statistical analysis of the clinical data. The normal distribution of measurement data was expressed as mean ± standard deviation, and the count data was expressed as a percentage. Differences between groups were compared using one-way ANOVA, nonparametric tests, *χ*^2^ tests, and paired *χ*^2^ tests. The Kappa consistency test was used to analyze the consistency between LUS and clinical diagnosis, CXR and chest CT in diagnosing CAP. All *P* values were two-sided and *P *< 0.05 was considered statistically significant. *Kappa value evaluation criteria*: A Kappa value of 0–0.20 was deemed very low consistency, a value of 0.21–0.40 was deemed average consistency, 0.41–0.60 represented medium consistency, 0.61–0.80 indicated high consistency, and a Kappa value of 0.81–1.00 was interpreted as very consistent.

The accuracy, sensitivity, specificity, positive predictive value (PPV), negative predictive value (NPV), and area under the receiver operating characteristic (ROC) curve of different models (area under curve of ROC, AUC) were calculated to assess the detection performance of the model. The accuracy, sensitivity, specificity, positive predictive value (PPV) and negative predictive value (NPV) were calculated to assess the classification performance of the model.

## Results

### General information of patients and comparison of baseline

**Figure 2:**
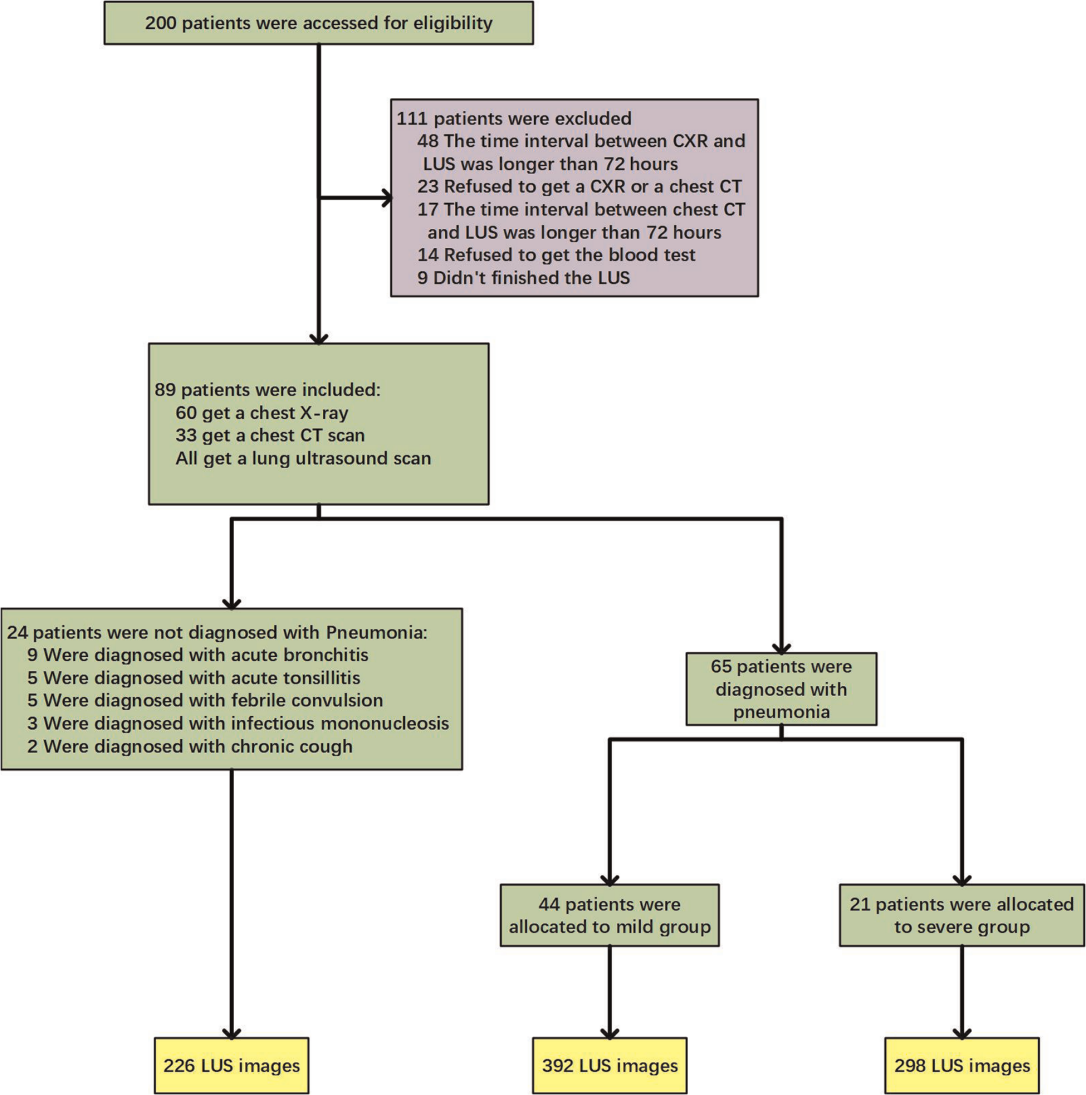
Trial flow diagram.

The clinical trial flow diagram was shown in Figure 2. Among the 200 children who met the inclusion criteria for this study, 111 patients were not included due to the time interval between CXR (48 cases) or chest CT (17 cases) and LUS longer than 72 h, or refusing imaging examinations (23 cases) or blood tests (14 cases), or being unable to cooperate in completing the LUS examination and taking images (9 cases). In the final 89 cases, 65 cases were clinically diagnosed with CAP, including 44 cases of mild disease and 21 cases of severe disease. Twenty-four cases were diagnosed with other diseases, such as acute bronchitis, acute tonsillitis, febrile convulsion, infection mononucleosis, etc. The LUS database constructed in this study was built from a total of 916 LUS images of the above-mentioned 89 children. A total of 64 cases were diagnosed as CAP by ultrasound, and 690 images of related lesions were obtained. Twenty-five cases were diagnosed as normal by ultrasound, and 226 images were obtained.

As displayed in Table 3, there was no statistical difference between the three groups in terms of gender, age, weight, allergy history, symptoms of upper respiratory tract infection, white blood cells, serum amyloid A, and procalcitonin (*P* > 0.05). Statistical differences were found among the three groups in terms of respiratory rate, days of fever before admission, general status, food refusal, moist rales, days of hospitalization, C-reactive protein, and interleukin-6 (*P* < 0.05) (Table 1). After pairwise comparison, the number of days of fever before hospitalization (*P* = 0.001), respiratory rate (adjusted *P* = 0.006), general status, food refusal, C-reactive protein (adjusted *P* = 0.03), interleukin-6 (adjusted *P* = 0.04), and the length of hospital stay (adjusted *P* = 0.01) were statistically different between the mild CAP group and the severe CAP group.

**Table 3:** Demographic and clinical characteristics of patients at baseline according to clinical diagnosis.

Variables	Clinical diagnosis (All patients, *n* = 89)			*P* value
	Non (*n* = 24)	Mild (*n* = 44)	Severe (*n* = 21)	
**General information**				
Gender, female, (*n* %)	12 (50)	20 (45.5)	5 (23.8)	.17
Age, years, median (IQR)	3 (1–6)	3 (2–4.75)	5 (3–6.5)	.07
Weight, kilograms, median (IQR)	15.2 (11.5–20)	15.6 (13.1–18)	21 (14.9–25.9)	.05
Allergy history, Positive, (*n* %)	3 (12.5)	7 (15.9)	5 (23.8)	.58
**Associated symptoms**				
Days of fever before hospitalization, average ± SD	2.8 ± 2.4	2.7 ± 2.3	4.9 ± 2.5	.002
Presentation of upper respiratory tract infection, Positive, (*n* %)	18 (75)	40 (90.9)	19 (90.5)	.196
General status, Well, (*n* %)	24 (100)	44 (100)	17 (81)	.002
Food refusal, Positive, (*n* %)	1 (6.7)	0 (0)	12 (57.1)	<.001
**Physical examination findings**				
Respiratory rate, median (IQR)	25 (22–29.5)	26 (24–28)	30 (27–32)	.004
Moist Rales, Positive, (*n* %)	0 (0)	18 (40.9)	9 (42.9)	0.001
**Laboratory findings, median (IQR)**				
WBC, 10^9^ cells/L	7 (5–11.2)	7.5 (5–10.8)	7 (4.5–8.5)	.36
CRP, mg/L	0.8 (0–8.8)	4.5 (0.5–14.8)	20.3 (4.5–35.8)	.001
SAA^a^, mg/L	57.4 (5.4–130.8)	48.8 (6.4–132.2)	108.0 (43.3–262.2)	.06
IL-6^b^, ng/L	9.2 (4–25.6)	8.1 (3.7–27.9)	34.3 (79–51.3)	.03
PCT^c^, ng/ml	0.1 (0.1–0.4)	0.1 (0.1–0.5)	0.1 (0.1–0.8)	.61
Hospital stay, median (IQR) or average ± SD	6 (4.3–6.8)	5 (5–6)	7 (6–7.5)	.02

IQR, interquartile range; SD, standard deviation; WBC, white blood cells; CRP, C-creative protein; SAA, serum amyloid A; IL-6, interleukin-6; PCT, procalcitonin. Non: diagnosed as other diseases other than pneumonia.

^a^ Obtained for 76 patients (non group: *n* = 21; mild group: *n* = 39; severe group: *n* = 16).

^b^ Obtained for 82 patients (non group: *n* = 21; mild group: *n* = 41; severe group: *n* = 20).

^c^ Obtained for 79 patients (non group: *n* = 21; mild group: *n* = 40; severe group: *n* = 18).

### Comparison of the value of LUS and clinical diagnosis, CXR and chest CT in the diagnosis of CAP

**Figure 3:**
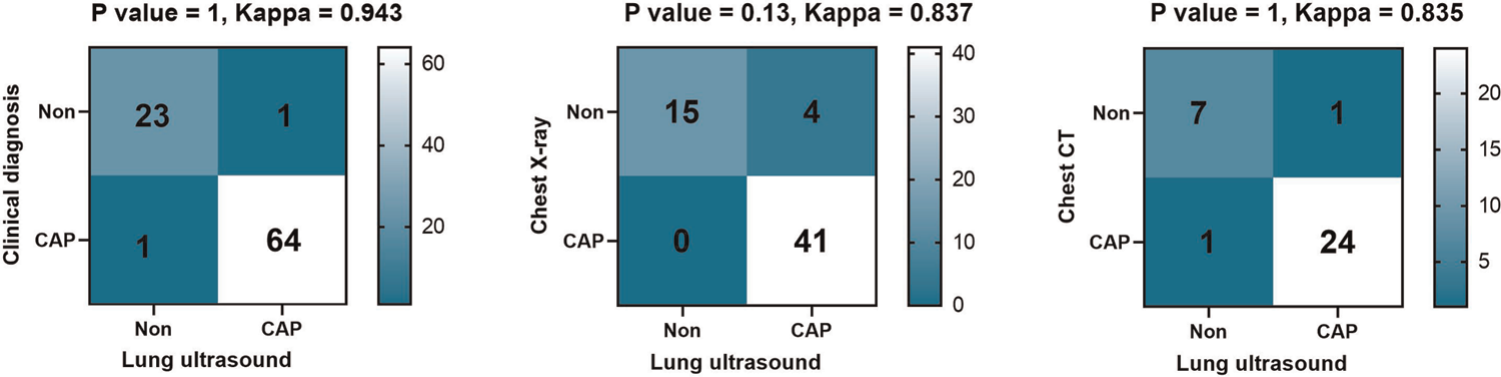
Clinical outcomes of hospitalized patients.

Among the 89 children, 65 were clinically diagnosed with CAP, 41 were diagnosed as CAP by CXR, and 25 were diagnosed as CAP by chest CT. Sixty-four cases were diagnosed as CAP by LUS; of these, one case was LUS-negative but diagnosed by chest CT as CAP, while one case was LUS-positive but the chest CT was negative. After calculation, the diagnostic accuracy, sensitivity and specificity of LUS emerged as 97.7%, 98.5% and 95.8% respectively. There was no statistical difference between LUS and clinical diagnosis, CXR and chest CT diagnosis of CAP (all *P* > 0.05), and the kappa values were 0.943, 0.837, and 0.835 respectively, which were shown in Figure 3.

### Efficacy evaluation of pre-trained CNN models for CAP diagnosis

**Figure 4:**
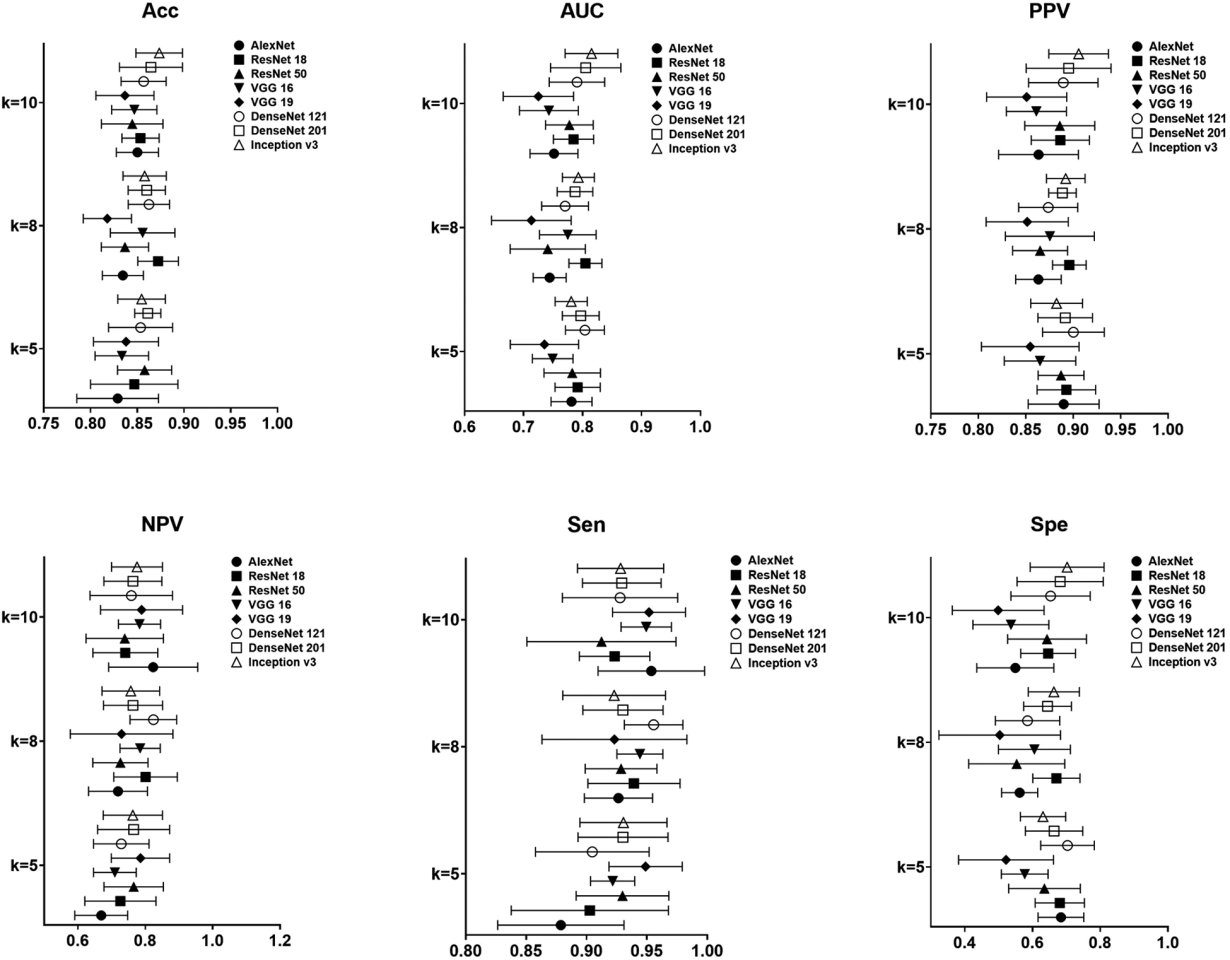
Result of performance metrics for the proposed 8 models in CAP detection for different k-folds cross validation.

In the task of identifying CAP in children by LUS, the metrics of accuracy, sensitivity, specificity, PPV, NPV, as well as AUC, for the proposed models were shown in Figure 4. It is clear that the performance metrics of the models changed when we increased the value of the fold. Our data achieved the highest accuracy of 0.87 ± 0.02, PPV of 0.90 ± 0.03, sensitivity of 0.92 ± 0.04 and AUC of 0.82 ± 0.04, respectively, in Inception v3 for 10-fold. The best NPV and specificity achieved in DenseNet 121, 0.70 ± 0.08 and 0.82 ± 0.07 by 5-fold and 8-fold. The average computational time of the 8 proposed modes for each image was 0.00173 s of AlexNet, 0.00183 s of VGG 16, 0.00205 s of VGG 19, 0.00283 s of Inception v3, 0.00203 s of ResNet 18, 0.00169 s of ResNet 50, 0.00150 s of DensetNet 121 and 0.00154 s of DenseNet 201.

### Efficacy evaluation of pre-trained CNN models for CAP severity classification

**Figure 5:**
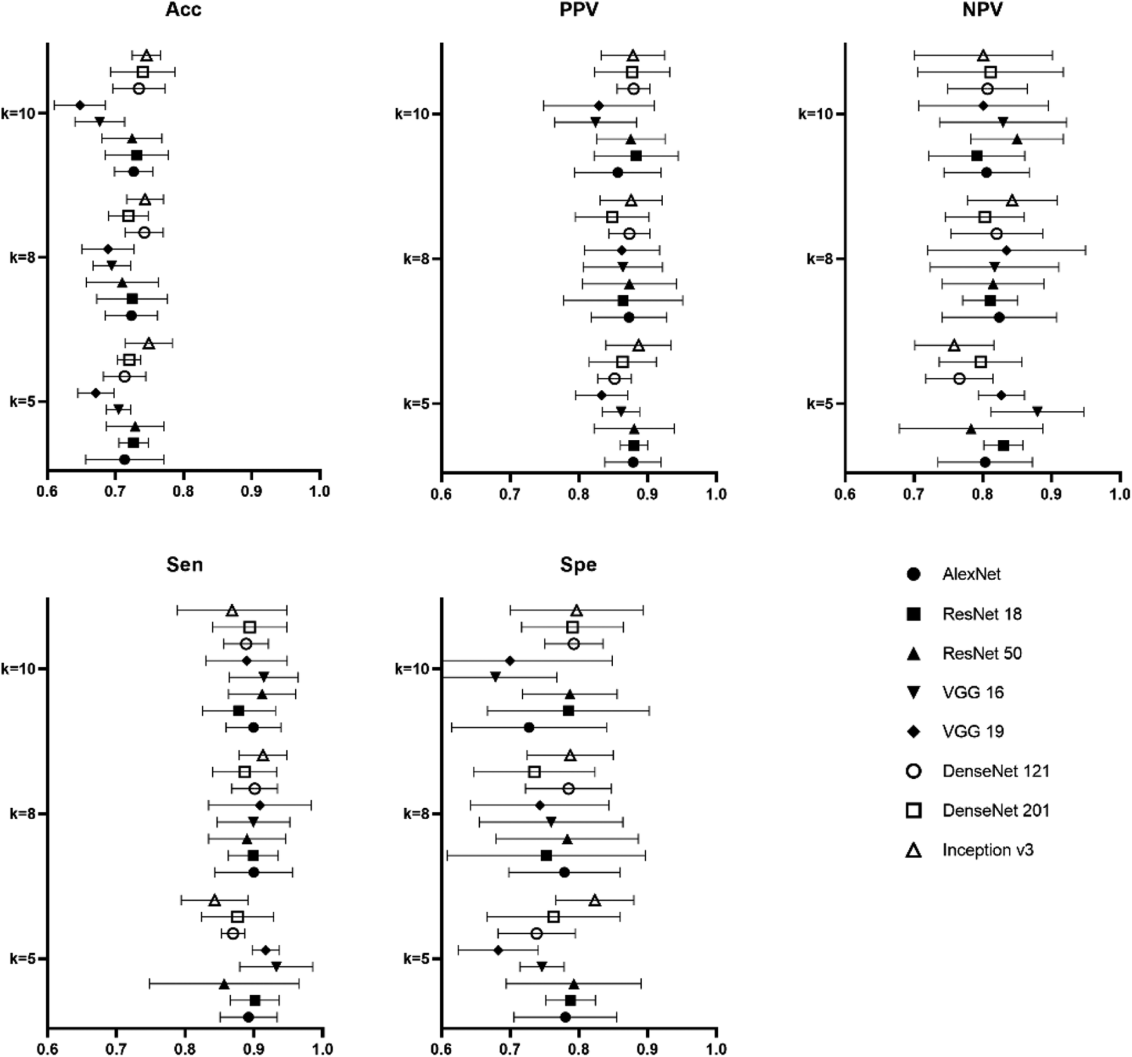
Result of performance metrics for the proposed 8 models in CAP severity classification for different k-folds cross validation.

Figure 5 was the result of the proposed 8 models in the task of degrading in children's CAP by LUS for different k-folds. On our data, highest accuracy, PPV and specificity of 0.75 ± 0.03, 0.89 ± 0.05 and 0.80 ± 0.10 were achieved in Inception v3 for 5-fold. As regards the best NPV of the pretrained models, VGG 16 achieved 0.88 ± 0.07 for 5-fold. The best sensitivity was also found in 0.93 ± 0.05 of VGG 16 for 5-fold.

## Discussion

### Principal findings

CAP is a common disease in children, especially in low-income countries, where the mortality rate of children in the first year with CAP is up to 50.13% of inpatient (24). Early and accurate diagnosis of CAP and judgment of the severity of the disease therefore have important clinical value (25). LUS can improve the shortcomings of CXR and chest CT with radioactivity. At the same time, machine learning models can enhance the efficiency of diagnostic methods and serve as a suitable alternative to the CXR and chest CT. No previous study has employed CNNs model for analyzing the LUS of children suspected with CAP. The study is a prospective case-control clinical trial and has established a standard LUS database. Our study suggests that LUS is a reliable method for diagnosing CAP in children, with comparable diagnostic capabilities to CXR and chest CT. The LUS-based AI recognition system has a good predictive ability for children's CAP based on our dataset. In the future, the CNNs method can be employed at diagnostic centers as a reliable method to detect CAP in children with high precision in the early stages of the disease, and achieve an automated and more efficient diagnosis.

### Comparison with other works

For children also have certain anatomical characteristics, such as a thin chest wall and small thorax, which are beneficial to ultrasound imaging, studies have shown that LUS can accurately diagnose pneumonia in children, and has the further advantages of no radiation, simplicity, dynamic observation, and repeatability. It can accordingly be used to replace pediatric CXR examinations, and has a diagnostic sensitivity and specificity no weaker than that of chest CT, which is a safe and feasible examination method. The diagnostic accuracy, sensitivity, and specificity of LUS in this study were found to be 97.7%, 98.5%, and 95.8%, respectively. LUS results were highly consistent with those of CXR and chest CT in the diagnosis of CAP (Kappa = 0.943, 0.837, 0.835), and were also consistent with previous findings. In a retrospective cohort study using chest CT as the gold standard, the sensitivity of LUS for diagnosing CAP was 90.6% and the accuracy was 66.1% (26). A meta-analysis further showed that the sensitivity of lung ultrasonography in diagnosing pneumonia in children was 98% with a specificity of 92% (27).

Ultrasonography relies more on the subjective judgment of the operator, meaning that inexperienced doctors may easily misdiagnose a condition. At the same time, the associated increase in work intensity may also increase the misdiagnosis rate of experienced doctors. Novice physicians often fail to correctly identify lesions and quantify their extent. AI technology is very well suited to solve these problems. Although no researchers have attempted to diagnose childhood pneumonia, at present, researchers have developed a CNN model to quantify the B-line of lung ultrasound in emergency dyspnea patients. The best CNN model established by the team had a sensitivity of 93% and a specificity of 96% when determining the presence of B-line (28). Gravina et al. (29) collected neonatal lung ultrasound images and videos to construct different CNN models to diagnose and differentially diagnose temporary tachypnea in neonates and neonatal respiratory distress syndrome, the highest accuracy of which was 87.8%. AI automated image analysis has advantages in its ability to assist doctors in improving the accuracy, efficiency and work intensity. In our study, the test results show that the operation time of every single image is extremely fast, which can significantly improve the diagnosis efficiency. According to our findings, Inception v3 achieved the highest diagnostic accuracy of 87%, which had similar diagnostic accuracy of lung ultrasound in other diseases, though the performance on the severity classification task is not satisfactory.

Nowadays, existing AI diagnosis systems designed for children's CAP are mainly based on clinical symptoms, physical examination and imaging examination of CXR. Abeyratne et al. (30) extracted the mathematical features of cough sounds through machine learning and used them to train a logistic regression classifier that classified cough sounds into pulmonary cough and non-pulmonary cough. The algorithm had a sensitivity of 94% and a specificity of 75% for the diagnosis of pneumonia. Other studies segmented the expiratory and inspiratory phases of breath sounds for parallel acoustic analysis (31). Sometimes patients don't have typical clinical manifestations. Mahomed et al. (32) analyzed and summarized the characteristics of children's CXR using an AI method and expressed the possibility of pneumonia through different colors; however, the AI's diagnostic sensitivity (76%) and specificity (80%) were low. Previous studies mostly used traditional machine learning methods; By contrast, our study uses LUS images to train the CNNs AlexNet, VGG 16, VGG 19, Inception v3, ResNet 18, ResNet 50, DenseNet 121 and DenseNet 201 using the TL method. After decades of technological development, the performance of AI diagnosis of CXR has been significantly improved (7–9), but this does not change the fundamental disadvantage of radiation. Since there is no public database of ultrasound images of children's pneumonia, prior to our study, no researchers have used LUS based on deep learning methods in the field of children's pneumonia. Upon the emergence of COVID-19, numerous CNNs techniques have been adopted to diagnose pneumonia of COVID-19. Table 4 lists some relevant studies. Our study detecting pneumonia in children had close accuracy to these studies of COVID-19. Consistently, our multi-class accuracy was slightly inferior to binary classification, which was the same as others.

**Table 4:** Studies using LUS based on CNNs for pneumonia of COVID-19.

Study (country)	Study objective	Population	Models used	Evaluation results
**Born et al. (Switzerland)** (12)	Pneumonia Classification COVID-19 Detection	1,103 images	POCOVID-Net	Accuracy = 0.89
				Sensitivity = 0.96
				Specificity = 0.79
				F1-score = 0.92.
**Born et al. (Switzerland)** (33)	Pneumonia Classification COVID-19 Detection	202 videos	optimized variations of VGG and NasNetmobile	Accuracy = 0.89
				Accuracy = 0.79
**Perera et al. (America)** (34)	COVID-19 Detection Pneumonia Classification	COVID-19: 84 video clips, Healthy: 75 video clips and Pneumonia: 53 video clips.	Proposed model	Accuracy = 0.87
				Sensitivity = 0.83
				Specificity = 0.97
				Accuracy = 0.90
				Sensitivity = 0.90
				Specificity = 0.98

Our study confirmed that LUS is a reliable method for diagnosing CAP in children. The CNN method can accurately identify CAP in children, which lays a theoretical foundation for future pediatric pulmonary AI-based ultrasound. However, this study still has certain limitations: (1) compared with the large number of datasets required for AI image recognition, the sample size used in this study is small. In the future, more images need to be collected to verify and test the model; (2) this study is a single-center study, in which a fixed number of doctors used a unified ultrasound instrument and probe to collect images. In future work, we will collect multi-center data for further research; (3) this AI system only observes LUS without clinical data. In the future, clinical data can be added on the basis of images; (4) due to the limited performance of single networks, an increasing number of studies have found that integration with other network models is an important development direction; (5) the clinical situation is always changeable, and the doctor still needs to combine other manifestations of the patient. In the future, we will improve AI diagnostic techniques that combine medical history and ultrasound signs, which may further increase the detection rate of other conditions. After all, AI cannot replace the doctor, if necessary, further examination is required.

## Conclusions

AI technology has great prospects in the promotion of LUS, especially in economically underdeveloped areas or in primary medical care, where they can improve the diagnostic accuracy of CAP. Using the automated diagnosis system for detecting children's CAP minimizes the time of image interpretation and consequently the possibility of delayed diagnosis for patients waiting at radiology centers. Furthermore, by increasing the number of images and comprehensively analyzing of multi-part images produced by the LUS and increasing the population size, better classification results for differentiating positive and negative cases can be expected in the future.

## Data Availability

The raw data supporting the conclusions of this article will be made available by the authors, without undue reservation.
